# The Relationship between Body Mass Index, Body Dissatisfaction and Mood Symptoms in Pregnant Women

**DOI:** 10.3390/jcm13082424

**Published:** 2024-04-21

**Authors:** Caterina Grano, Mariacarolina Vacca, Caterina Lombardo

**Affiliations:** Department of Psychology, Sapienza University of Rome, 00185 Rome, Italy

**Keywords:** pregnancy, body dissatisfaction, depression, anxiety, body mass index

## Abstract

**Background**: High body mass and adiposity during pregnancy can contribute to psychological distress, and body dissatisfaction may be a potential underlying mechanism of this association. Objective. This study aimed to evaluate the mediational role of body dissatisfaction in the relationship between body mass index (BMI) and depressive and anxious symptoms, respectively. **Methods**: Given the cross-sectional design of this study, two alternative models were investigated, positing that BMI was related to depressive (Model 1a) and anxious symptoms (Model 2a), which, in turn, predicted body dissatisfaction. Seventy-two pregnant women in the third trimester of pregnancy completed the Body Image Disturbance Questionnaire, the Beck Depression Inventory-II and the State-Trait Anxiety Inventory, as well as a demographic form assessing their BMI. **Results**: As hypothesized, body dissatisfaction mediated the relationship between BMI and psychopathological symptoms. Moreover, the alternative models of reverse mediation were also significant, suggesting that psychopathological symptoms mediated the relationship between BMI and body dissatisfaction. Findings from both the hypothesized and alternative models suggested that, on the one hand, higher distress symptoms associated with body dissatisfaction would result from high BMI and, on the other hand, that body dissatisfaction may result from the effect of BMI on distress symptoms. **Conclusions**: The present study suggests that body image theory and practice should be implemented by the inclusion of evidence-based clinical interventions for promoting psychological well-being during the antenatal period.

## 1. Introduction

During pregnancy, a series of physiological and psychological changes take place in a short period of time [1] and affect women, both physically and mentally [2]. In this complex phase, women may experience the rapid body changes that come with motherhood as a dramatic event and feel a profound dissatisfaction with their own bodies [3]. For instance, from the first trimester of pregnancy [4], women experience a significant increase in their body mass index (BMI), resulting in a strongly perceived discrepancy between their actual body and the body ideals imposed by society and the media. Some studies report that women adapt to changes in their bodies and report larger ideal body sizes as pregnancy progresses [5]. Other evidence shows that pregnant women desire to have a smaller body size [6] and reported a negative “body schema” when compared with non-pregnant women [7]. BMI was found to be associated with body dissatisfaction during pregnancy, as high levels of body weight and shape concerns were more prevalent among pregnant women with a high BMI [8].

Considering mental health symptoms, the role of BMI is well established in pregnancy [9]. Prevalence estimates for depression and anxiety are approximately 20.7% and 15.2%, respectively [10,11,12]. A strong correlation between high pre-pregnancy BMI and the risk of developing depression during pregnancy has been reported, with even small BMI increases within the range of ‘normal weight’ being associated with a higher likelihood of major depression [9,10]. Studies evidenced that pre-pregnancy BMI predicted anxiety during pregnancy and was positively associated with post-partum levels of anxiety [7,13].

In investigating which psychological variables may contribute to explaining the association between BMI and mental health in pregnancy, many authors suggested that negative body image had a role in accounting for this relationship. A plethora of studies concur with this hypothesis, highlighting the potential mediating role of individual concerns and dissatisfaction regarding weights and shapes in mediating this association [14,15].

This mediation hypothesis stems from the repercussions of the internalization of societal ideals regarding weight, where discontent with one’s body can lead to a deteriorated psychological well-being [16]. This conceptual model has been evaluated in the general and community-based populations [14,15,17], while results among pregnant women are limited. One previous study [18] found that body image concerns mediated the total effect of weight change on depressive symptoms in pregnant women over time. The authors suggested that the mediation effect of body image was greater among women with higher pre-pregnancy BMI. This theoretical model could be explained by considering that the significant elevation of body mass and adiposity during pregnancy [8] can lead women to experience a deviation from the thin ideal imposed by the media and society, which, in turn, contributes to psychological distress [1]. In line with this perspective, the present study aims to evaluate this conceptual framework in a sample of first-trimester pregnant women. In light of the fact that much of the research on mediators of the BMI–mental health link has primarily focused on depression, it is crucial to explore connections with anxiety, considering the well-known association of BMI [9] and negative body image [19] with anxiety symptoms. Therefore, two mediation models were tested wherein BMI predicted depressive and anxious symptoms through body dissatisfaction. Since the cross-sectional nature of the present work did not permit us to make predictions regarding the sequential relationship between these variables, an alternative model for each outcome was explored. These models posited that BMI was related to depressive and anxiety symptoms [9], which, in turn, predicted body dissatisfaction [20].

## 2. Materials and Methods

### 2.1. Study Design and Setting

The study had a cross-sectional design. Inclusion criteria were limited to women in the first trimester of pregnancy who were proficient in Italian. Participation was voluntary, and no financial compensation was offered. The study was presented as aiming to evaluate the association between several pregnancy-related variables. Participants were approached between February 2018 and October 2020 by three master’s students of Psychology courses from an Italian university to complete an online self-report survey. The typical channels of recruitment were friends, acquaintances, private maternity centers, schools, family associations and diagnostic clinics. Following an explanation of the study procedure and the signing of informed consent forms, participants were invited to complete an online questionnaire. The protocol of the present study was approved by the Institutional Review Board (prot. n. 0000102, approval date: 24 January 2018). All procedures performed in studies involving human participants were conducted in accordance with the ethical standards of the institutional and national research committee and with the 1964 Declaration of Helsinki and its later amendments or comparable ethical standards. The study data entry and management systems used by the researchers were secured and password-protected. The principal investigator had access to the collected data and study documents. If participants requested to leave the study, no additional data were collected, and any existing data previously collected were destroyed. Individual participants and their research data were identified by a unique study identification number.

### 2.2. Participants

Assuming large effect sizes and with the power set at 0.80, a sample size of 70 people was suggested by 5000 Monte Carlo simulations [21].

Seventy-two pregnant women (age range: 21–42 years; mean age = 32.110 years, SD = ±4.586) were included in the present study. No data were missing.

### 2.3. Measures

#### 2.3.1. BMI

Women were requested to report their height (m) and weight (Kg) before the current pregnancy. BMI (kg/m^2^) was computed in the statistical analysis process.

#### 2.3.2. Body Dissatisfaction

The 7-item Body Image Disturbance Questionnaire (BIDQ) [22] was employed to assess the levels of body dissatisfaction, preoccupation, dysfunction and distress. The Cronbach alpha reliability [18] for this scale was good (α = 0.678). The scale evaluates preoccupations, distress and impairment in social or occupational functioning related to participants’ body appearance. An example item is: “Are you concerned about the appearance of some part(s) of your body, which you consider especially unattractive?” Items were rated on a five-point Likert scale ranging from 1 (“not at all concerned”) to 5 (“extremely concerned”). The total score is computed as the mean of the items, with a higher score indicating higher levels of body image disturbance.

#### 2.3.3. Depressive Symptoms

The 21-item Beck Depression Inventory-II (BDI-II) [23] was used to examine levels of depressive symptoms. The BDI-II consists of 21 items assessing the severity of typical depression symptoms over the prior two weeks, according to the Diagnostic and Statistical Manual of Mental Disorders—Fourth Edition (DSM-IV) [24]. Each item is rated on an intensity scale of 0–3 [23]. An example item is “I can’t get any pleasure from the things I used to enjoy”. High scores indicate high depression, and the total continuous score ranges from 0 (low) to 63 (high). BDI-II scores lower than 13 suggest a low risk of severe depression, whereas scores of 13 or above are indicative of a high risk for severe symptoms. The internal reliability found in the present research was high (α = 0.836).

#### 2.3.4. Anxiety Symptoms

The 20-item trait anxiety scale of the State-Trait Anxiety Inventory [25] was employed to assess the general level of anxiety (i.e., how the subject ‘usually’ feels). Items were on a 4-point scale (1 = Rarely to 4 = Almost always), and the internal consistency was good (α = 0.782). The total score ranges from 20 to 80, with higher scores indicating more severe anxiety. More specifically, scores lower than 40 indicate a low risk of severe anxiety, whereas scores equal to or higher than 40 indicate an elevated risk of anxiety [26]. This instrument is the most widely used tool in research on anxiety in women during pregnancy [27].

#### 2.3.5. Analytic Strategy

After assessing the normality of the data, descriptive statistics and correlations between the variables were examined. The variance inflation factor (VIF) and multicollinearity tolerance (TOL) were estimated to test multicollinearity between all the variables included in the mediation models. A problem with multicollinearity is indicated by a TOL below 0.2 and a VIF above 10 [28].

Mediation analysis was performed to test the hypotheses included in the proposed conceptual framework. Direct and indirect effects were examined with the Process macro for SPSS 25 with confidence intervals for the indirect effect determined by bootstrapping with 5000 iterations [29]. Separate analyses were conducted for each outcome variable, with variables being standardized before the analysis. As recommended by Kenny [30], in order to strengthen support for a mediational model tested using cross-sectional data, an alternative model was assessed involving reverse mediation for each of the two models tested.

In the first model (Model 1), the mediation role of body dissatisfaction on the relationship between the BMI before pregnancy and depressive symptoms, controlling for primiparous motherhood, was investigated. In the alternative model, body dissatisfaction and depressive symptoms were switched, such that body dissatisfaction served as the dependent variable and depressive symptoms served as the mediation variable.

In the second model (Model 2), the mediation role of body dissatisfaction on the relationship between the BMI before pregnancy and anxiety symptoms, controlling for primiparous motherhood, was investigated. In the alternative model, body dissatisfaction and anxiety symptoms were switched, such that body dissatisfaction served as the dependent variable and anxiety symptoms served as the mediation variable.

## 3. Results

### 3.1. Preliminary Analyses

Descriptive statistics and correlations among the variables included in the models are presented in Table 1 and Table 2, respectively. No multicollinearity problems were found for any of the variables, which showed TOL and VIF values of collinearity within acceptable levels. With regard to the level of anxiety scores, 43.7% showed no anxiety/mild anxiety, 50.7% reported moderate anxiety and 5.6% had severe anxiety symptoms. On the other hand, the majority of participants (78.9%) did not report depressive symptoms, whereas 21.1% showed clinically relevant symptoms, from mild (16.9%) to severe (1.4%).

### 3.2. Mediation Testing

**Figure 1:**
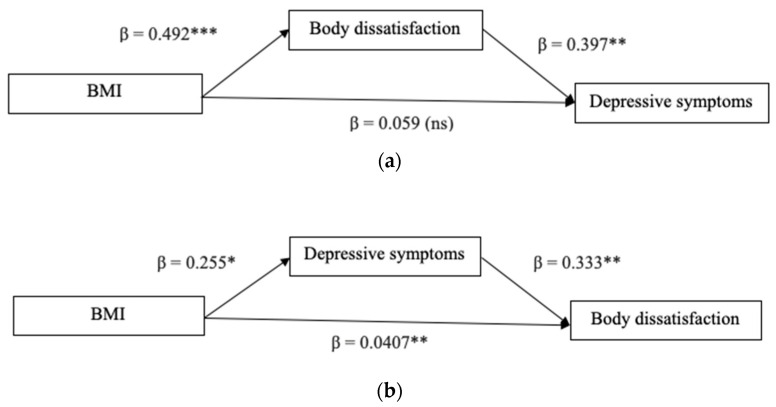
(**a**) Hypothesized model; (**b**) Alternative model. Note. Standardized coefficients are displayed. * *p* < 0.05. ** *p* < 0.01. *** *p* < 0.001.

Path coefficients for Model 1 are presented in Figure 1a. As hypothesized, body dissatisfaction mediated the relationship between BMI and depressive symptoms, as indicated by the indirect effect (β = 0.195, SE = 0.063, 95% CI [0.089, 0.334], *p* < 0.05). The direct effect was not significant (β = 0.059, SE = 0.127, 95% CI [−0.194, 0.322]), thus indicating a full mediation. No significant effect for the covariate was found. The overall amount of variance explained in depressive symptoms was significantly different from zero, R^2^ = 0.202, F (3, 67) = 5.685, *p* < 0.01. An alternative model (Figure 1b) was run to provide the specificity of the findings with the presence of depressive symptoms as the indirect predictor and body dissatisfaction as the dependent variable. Depressive symptoms mediated the relationship between BMI and body dissatisfaction, as indicated by the indirect effect (β = 0.085, SE = 0.047, 95% CI [0.003, 0.196], *p* < 0.05). Differently from the original Model 1, in this model, the direct effect was significant (β = 0.407, SE = 0.105, 95% CI [0.197, 0.618], *p* < 0.01), thus indicating a partial mediation. No significant effect for the covariate was found. The overall amount of variance explained in depressive symptoms was significantly different from zero, R^2^ = 0.232, F (3, 67) = 10.28, *p* < 0.001.

**Figure 2:**
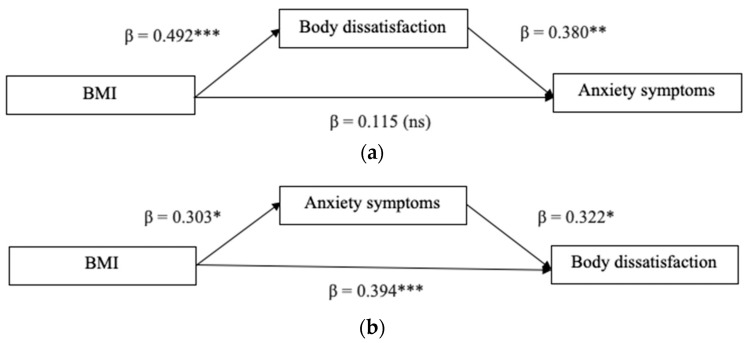
(**a**) Hypothesized model; (**b**) Alternative model. Note. Standardized coefficients are displayed. * *p* < 0.05. ** *p* < 0.01. *** *p* < 0.001.

Model 2 was tested in order to examine the mediation role of body dissatisfaction in the relationship between BMI and anxiety symptoms. The results are displayed in Figure 2a. A significant indirect effect was found (β = 0.187, SE = 0.066, 95% CI [0.077, 0.338], *p* < 0.05). The direct effect of BMI on anxiety symptoms was not significant (β = 0.115, SE = 0.127, 95% CI [−0.138, 0.369]), suggesting that a full mediation occurred. No significant effect for the covariate was found. The model explained the 8% variance of anxiety symptoms (R^2^ = 0.089, F (2, 68) = 3.33, *p* < 0.05).

The relative alternative model (Figure 2b) was examined to test the mediation role of anxiety symptoms in explaining the association between BMI and body dissatisfaction. The results showed a significant indirect effect (β = 0.097, SE = 0.049, 95% CI [0.020, 0.213], *p* < 0.05) and a significant direct effect (β = 0.394, SE = 0.107, 95% CI [0.180, 0.609], *p* < 0.01), thus indicating a partial mediation. No significant effect for the covariate was found. The model explained a high percentage of variance in body dissatisfaction (R^2^ = 0.326, F (3, 67) = 10.83, *p* < 0.001).

## 4. Discussion

The main goal of this study was to examine the proposed relationship between BMI and anxiety and depressive symptoms among first-trimester pregnant women and to establish if negative body image accounted for this relationship. The alternative models were also tested, with depression and anxiety as mediators and body dissatisfaction as an outcome.

First, descriptive statistics on anxiety and depression mean scores reflected findings of previous studies (e.g., [31,32,33,34,35]). More specifically, around half of the sample reported moderate anxiety symptoms, supporting evidence of high prevalence rates of a moderate degree of general anxiety during the first trimester of pregnancy [33]. Regarding levels of depression, over 75% reported no symptoms, as indicated by previous authors [35]. Moreover, depression mean scores were somewhat lower than this previous evidence. In the present study, women were involved when they were in the first trimester of pregnancy. Since changes in life associated with the birth process (e.g., problematic sleep patterns [36]) are not imminent in this period of gestation [37], women may be less prone to experience depression at this stage. Some authors suggest that stressors occurring during pregnancy (e.g., physical problems) may have a higher impact on antenatal symptoms of depression than those occurring before pregnancy [38]. Future studies should assess longitudinal changes in depression levels across pregnancy stages according to adverse life events and stressors experienced.

Mediation analyses showed that a higher BMI predicted greater depressive symptoms through its incremental impact on body dissatisfaction. A similar pattern of relationships occurred when considering anxiety symptoms. Namely, higher BMI was associated with a greater likelihood of reporting body image concerns, which, in turn, were related to high anxiety. Overall, these findings indicated that body dissatisfaction may worsen in higher BMI pregnancies and that this effect may induce more depressed and anxious feelings. The results on depression are consistent with previous evidence suggesting that body image concerns mediate the effect of weight change on depressive symptoms in pregnant women over time [18]. However, in the hypothesized mediation model, the results showed that the direct effect of BMI on depression and anxiety is non-significant, indicating that a full mediation occurred. This evidence expands the literature supporting the positive association between BMI and mood symptoms [14] by suggesting the existence of an underlying mechanism explaining how women with higher BMI have a greater risk of developing distress.

Because the study design was cross-sectional, it was plausible that alternative mediation models also fit the data. Therefore, two alternative models whereby the mediating variable (i.e., body dissatisfaction) and the outcome variable (i.e., depression, anxiety) were switched were tested.

The results support the mediation with these alternative models. Namely, depressive symptoms (i.e., Model 1a) and anxiety symptoms (i.e., Model 2a) partially mediated the impact of BMI on body dissatisfaction. However, the confidence intervals for the alternative models were closer to zero as compared to those observed in the hypothesized model. As such, there was stronger support for the hypothesized model, which is more consistent with previous work on the relationship between BMI and distress in pregnant women [18].

Taken together, the results from both the hypothesized and alternative models suggested that, on the one hand, higher distress symptoms may be associated with body dissatisfaction, which would result from high BMI [18], while on the other hand, body dissatisfaction may result from distress symptoms [20], which, in turn, depend on BMI. Evidence from the first model substantiated the literature on body image concerns, asserting that they can substantially influence the risk of developing mood problems for women who begin their pregnancies with higher body weights [18]. The correlation observed between body dissatisfaction and depressive symptoms reinforces the “thinness ideal theory”, which suggests that being overweight or obese heightens body dissatisfaction, which, in turn, results in weight-related concerns or pressures that contribute to mental distress symptoms [20]. However, the results of the alternative model suggested that body dissatisfaction may follow depressive symptoms, as previously observed [20]. These findings substantiate evidence in the literature that depression may involve negative appraisals of the self, including body image concerns [39,40]. Indeed, previous prospective results supported that depression predicted body dissatisfaction in late pregnancy and post-partum periods [19].

### 4.1. Limitations

The results of the study need to be considered within the context of its limitations. First, the use of self-report questionnaires did not permit the exclusion of social desirability in the responses given by participants. Future studies should employ more informative instruments of assessment to evaluate body image concerns and mental distress symptoms. In this regard, qualitative and interview-based methods [41] could be integrated with objective approaches in further research to provide more comprehensive data. Moreover, the use of self-reported pre-pregnancy weight and self-reported height measurements to calculate the pre-pregnancy BMI might have also affected the results. Although self-reported weight is largely concordant with measured pre-pregnancy weight [42], future studies should include an anthropometric assessment of BMI.

Second, the cross-sectional nature of the data precludes the ability to draw causal inferences. Although testing of a plausible alternative model lends some additional support to the findings in our study, longitudinal models are needed to validate the directional pathways. Additionally, ecological momentary assessments could be used to determine the stability or fluctuations of irritability over varying periods of pregnancy [43].

Lastly, the current sample exhibited heterogeneity in terms of depression, as 21.1% showed clinically relevant symptoms. Future works should evaluate whether the mediation models proposed in this study are statistically meaningful among pregnant women with and without depressive manifestations.

### 4.2. Conclusions

Despite the aforementioned limitations, this study may have several promising treatment-related implications. First, women with high body image concerns may require particular support during pregnancy in clinical settings. Clinicians need to understand how body dissatisfaction during pregnancy can negatively impact women’s mental health, as well as how psychological distress may exacerbate body dissatisfaction during pregnancy [35]. Body image theory and practice should be implemented by the inclusion of evidence-based clinical interventions to promote psychological well-being during the antenatal period [1]. This issue is relevant, especially considering that pregnancy-related changes in body image are still disregarded by healthcare practitioners working with pregnant women [36]. Moreover, the results of the present investigation may indicate that antenatal education programs should consider pregnant women with high BMI as more vulnerable to body image concerns and mental distress. Notwithstanding, some authors suggest that interventions to improve body image could benefit all women, regardless of their weight status [15]. Therefore, in accordance with recent observations [37], assessing body image during pregnancy should be regarded as a crucial task for pre-natal care professionals.

Findings from the hypothesized model suggested that higher distress symptoms were positively associated with BMI through body dissatisfaction. Also, the alternative model revealed that body dissatisfaction may result from the effect of BMI on distress symptoms. This evidence sheds new light on the role of BMI as a well-established construct for examining mental health in pregnancy. The causal relationships underlying these constructs in pregnancy are deferred to future work.

## Figures and Tables

**Table 1:** Descriptive statistics (N = 72).

Variables	BMI	BIDQ	BDI-II	STAI
M	24.372	12.958	9.859	42.338
SD	4.799	4.908	5.436	8.148
Range	17.58–38.67	2–28	0–31	30–69
α	-	0.678	0.836	0.782

Note. Abbreviations: M, mean; SD, standard deviation; BMI, body mass index; BIDQ, Body Image Disturbance; Questionnaire; BDI-II, Beck Depression Inventory-II; STAI, State-Trait Anxiety.

**Table 2:** Bivariate correlations included in the present study and descriptive statistics (N = 72).

Variables	1	2	3	4	5
Anxiety symptoms	−				
Depressive symptoms	0.661 *	−			
Body dissatisfaction	0.436 **	0.424 **	−		
BMI	0.285 *	0.217	0.481 **	−	
Motherhood status	−0.031	−0.136	0.009	0.186	−

Note. * *p* < 0.05. ** *p* < 0.01. Primiparous motherhood status: 1 = yes; 2 = no.

## Data Availability

The data presented in this study are available on request from the corresponding author.
